# In Vivo Protective Effects of Nootkatone against Particles-Induced Lung Injury Caused by Diesel Exhaust Is Mediated via the NF-κB Pathway

**DOI:** 10.3390/nu10030263

**Published:** 2018-02-26

**Authors:** Abderrahim Nemmar, Suhail Al-Salam, Sumaya Beegam, Priya Yuvaraju, Naserddine Hamadi, Badreldin H. Ali

**Affiliations:** 1 Department of Physiology, College of Medicine and Health Sciences, United Arab Emirates University, P.O. Box 17666 Al Ain, UAE; sumayab@uaeu.ac.ae (S.B.); priyay@uaeu.ac.ae (P.Y.); 2 Department of Pathology, College of Medicine and Health Sciences, United Arab Emirates University, P.O. Box 17666 Al Ain, UAE; suhaila@uaeu.ac.ae; 3 Department of Pharmacology, College of Medicine and Health Sciences, United Arab Emirates University, P.O. Box 17666 Al Ain, UAE; hamadinasro@uaeu.ac.ae; 4 Department of Pharmacology and Clinical Pharmacy, College of Medicine & Health Sciences, Sultan Qaboos University, P.O. Box 35, Muscat 123, Al-Khod, Oman; alibadreldin@hotmail.com

**Keywords:** diesel exhaust particles, nootkatone, airway resistance, Lung, oxidative stress, inflammation, NF-κB

## Abstract

Numerous studies have shown that acute particulate air pollution exposure is linked with pulmonary adverse effects, including alterations of pulmonary function, inflammation, and oxidative stress. Nootkatone, a constituent of grapefruit, has antioxidant and anti-inflammatory effects. However, the effect of nootkatone on lung toxicity has not been reported so far. In this study we evaluated the possible protective effects of nootkatone on diesel exhaust particles (DEP)-induced lung toxicity, and the possible mechanisms underlying these effects. Mice were intratracheally (i.t.) instilled with either DEP (30 µg/mouse) or saline (control). Nootkatone was given to mice by gavage, 1 h before i.t. instillation, with either DEP or saline. Twenty-four hours following DEP exposure, several physiological and biochemical endpoints were assessed. Nootkatone pretreatment significantly prevented the DEP-induced increase in airway resistance in vivo, decreased neutrophil infiltration in bronchoalveolar lavage fluid, and abated macrophage and neutrophil infiltration in the lung interstitium, assessed by histolopathology. Moreover, DEP caused a significant increase in lung concentrations of 8-isoprostane and tumor necrosis factor α, and decreased the reduced glutathione concentration and total nitric oxide activity. These actions were all significantly alleviated by nootkatone pretreatment. Similarly, nootkatone prevented DEP-induced DNA damage and prevented the proteolytic cleavage of caspase-3. Moreover, nootkatone inhibited nuclear factor-kappaB (NF-κB) induced by DEP. We conclude that nootkatone prevented the DEP-induced increase in airway resistance, lung inflammation, oxidative stress, and the subsequent DNA damage and apoptosis through a mechanism involving inhibition of NF-κB activation. Nootkatone could possibly be considered a beneficial protective agent against air pollution-induced respiratory adverse effects.

## 1. Introduction

Particulate air pollution consists of a complex mixture of solid and liquid particles of organic and inorganic substances suspended in the air [1]. Fine particulate matter and particulate matter with an aerodynamic diameter of less than 2.5 μm (PM_2.5_) has been associated with significant adverse health effects, including increases in morbidity and mortality, particularly in the cardiovascular and respiratory systems [1,2].

Exhaust from diesel powered vehicles is one of the main sources of diesel exhaust particles (DEP), one of the major constituents of PM_2.5_ and nanoparticles (diameter ≤ 0.1 µm) in urban areas [1,3]. These nanoparticles are characterized by their small size, allowing them to penetrate deeply into the respiratory tract, and their high alveolar deposition and large surface area, permitting the carrying of significant amounts of toxic compounds, (e.g., hydrocarbons and metals) [3]. These particles were reported to cause pulmonary oxidative stress and inflammation, and alter cardiac autonomic function in humans and experimental animals [1,3,4]. Acute controlled exposure to emissions from diesel engines in healthy human volunteers has been shown to cause a decrease in lung function, and induce respiratory irritation, inflammation, oxidative stress, and adverse cardiovascular effects [5,6,7]. Likewise, experimental studies in mice and rats reported the occurrence of lung inflammation, oxidative stress and increase in airway resistance [8,9,10].

Nootkatone, a sesquiterpene, is a recognized bioactive compound, isolated from the rhizomes of *Cyperus rotundus* [11]. Nootkatone is also naturally found in grapefruit oil [11,12]. It has been reported that nootkatone has several pharmacological properties, such as antiseptic, antioxidant, and antiallergic activities [11,13,14].

Since respiratory toxicity, related to DEP, involves oxidative stress and inflammation [6,7,8,15], and nootkatone has palliative effects against some experimental diseases involving inflammation and oxidative stress [11,13], we considered that it was relevant to assess the possible ameliorative effects of nootkatone on DEP-induced lung injury and the mechanisms underlying these effects in mice. This is the first study on such an interaction.

## 2. Material and Methods

### 2.1. Animals and Treatments

This project was reviewed and approved by the Institutional Review Board of the United Arab Emirates University, College of Medicine and Health Sciences, and experiments were performed in accordance with protocols approved by the Institutional Animal Care and Research Advisory Committee.

### 2.2. Diesel Exhaust Particles (DEP) and Animal Treatments

The DEP (SRM 2975) were obtained from the National Institute of Standards and Technology (NIST, Gaithersburg, MD, USA), and were suspended in sterile saline (NaCl 0.9%), containing Tween 80 (0.01%). To minimize aggregation, particle suspensions were sonicated (Clifton Ultrasonic Bath, Clifton, NJ, USA) for 15 min and vortexed before their dilution, and prior to intratracheal (i.t.) administration. Control animals received saline containing Tween 80 (0.01%). These particles were previously analysed by transmission electron microscopy, and shown to have a substantial amount of ultrafine (nano) sized particle aggregates, and larger particle aggregates [16].

Animals and treatments: BALB/C mice (Taconic Farms Inc., Germantown, NY, USA), weighing 20–25 g, were housed in light (12-h light: 12-h dark cycle) and temperature-controlled (22 ± 1 °C) rooms. They had free access to commercial laboratory chow and were provided with tap water ad libitum.

The pulmonary exposure to DEP was achieved by i.t. administration [17]. Mice were first anesthetized with sodium pentobarbital [60 mg/kg, intraperitoneal (i.p.)] and placed supine with an extended neck on an angled board. A Becton Dickinson 24 Gauge cannula was inserted via the mouth into the trachea. Either the DEP suspension (30 μg/mouse) [17] or vehicle was instilled i.t. (100 µL) via a sterile syringe and followed by an air bolus of 100 µL. Nootkatone was administered by gavage (90 mg/kg), 1 h before exposure to either DEP or vehicle. The dose of nootkatone used here has been chosen from our pilot experiments, which showed its effectiveness in preventing cellular infiltration in the lung as compared with 10 and 30 mg/kg (Supplementary Table S1). The animals were randomly divided into four equal groups and were treated as follows:

- Group 1: Normal saline administered by gavage 1 h prior to pulmonary exposure to vehicle;
- Group 2: Normal saline administered by gavage 1 h prior to pulmonary exposure to DEP (30 μg/mouse);
- Group 3: Nootkatone (90 mg/kg) administered by gavage 1 h prior to pulmonary exposure to vehicle;
- Group 4: Nootkatone (90 mg/kg) administered by gavage 1 h prior to pulmonary exposure to DEP (30 μg/mouse).

Twenty-four hours after the pulmonary exposure to either DEP or vehicle, airway hyperresponsiveness and lung inflammation, oxidative stress, DNA damage, apoptosis and nuclear factor-kappa-B (NF-κB) activation were assessed.

### 2.3. Airway Reactivity to Methacholine

Airway hyperreactivity responses were measured using a forced oscillation technique (FlexiVent, SCIREQ, Montreal, QC, Canada). Airway resistance (R) was assessed after increasing exposures to methacholine. Mice were anesthetized with an intraperitoneal injection of pentobarbital (70 mg/kg). The trachea was exposed and an 18-gauge metal needle was inserted into the trachea. Mice were connected to a computer-controlled small animal ventilator and quasi-sinusoidally ventilated, with a tidal volume of 10 mL/kg at a frequency of 150 breaths/min and a positive end-expiratory pressure of 2 cm H_2_O, to achieve a mean lung volume close to that observed during spontaneous breathing. After measurement of a baseline, each mouse was challenged with methacholine aerosol, generated with an in-line nebulizer and administered directly through the ventilator for 5 s, with increasing concentrations (0, 0.625, 2.5, 10 and 40 mg/mL). Airway resistance (R) was measured using a “snapshot” protocol each 20 s for 2 min. The mean of these five values was used for each methacholine concentration, unless the coefficient of determination of a measurement was smaller than 0.95. For each mouse, R was plotted against methacholine concentration (from 0 to 40 mg/mL) [18,19].

### 2.4. Collection and Analysis of Bronchoalveolar (BAL) Fluid

The collection and analysis of BAL was performed, in separate animals, according to a previously described method [8,18,20,21]. In brief, mice were sacrificed with an overdose of sodium pentobarbital after either DEP or saline administration, with or without nootkatone treatment. The trachea was cannulated and lungs were lavaged three times with 0.7 mL (a total volume of 2.1 mL) of sterile NaCl 0.9% solution. The recovered fluid aliquots were pooled. No difference in the volume of collected fluid was observed between the different groups. BAL fluid was centrifuged (1000× *g* 10 min, 4 °C). Cells were counted in a Thoma hemocytometer after resuspension of the pellets and staining with 1% gentian violet. The cell differentials were microscopically performed on cytocentrifuge preparations fixed in methanol and stained with Diff Quick (Dade, Brussels, Belgium).

### 2.5. Histology

In separate groups of animals, the lungs were excised, washed with ice-cold saline, blotted with filter paper and weighed. Each lung was dissected, casseted and fixed directly in 10% neutral formalin for 24 h, which was followed by dehydration with increasing concentrations of ethanol, clearing with xylene and embedding with paraffin. Three-μm sections were prepared from paraffin blocks and stained with haematoxylin and eosin. The stained sections were evaluated by the histopathologist, who participated in this project, using light microscopy [8].

### 2.6. Measurement of 8-Isoprostane, Reduced Glutathione (GSH), Total Nitric Oxide (NO) and Tumor Necrosis Factor (TNFα) in Lung Homogenates

Following the exposure to either DEP or saline, with or without nootkatone pretreatment, individual mice were sacrificed by an overdose of sodium pentobarbital, and their lungs were quickly collected and rinsed with ice-cold PBS (pH 7.4) before homogenization, as described before [9]. The homogenates were centrifuged for 10 min at 3000× *g* to remove cellular debris, and the supernatants were used for further analysis [9]. Protein content was measured by Bradford’s method. Concentrations of 8-isoprostane were determined using an ELISA Kit (Cayman Chemicals, Ann Arbor, MI, USA) [22]. The concentrations of TNFα were determined using ELISA Kits (Duo Set, R & D Systems, Minneapolis, MN, USA). Measurement of GSH concentrations was carried out according to the method described for the commercially available kit (Sigma-Aldrich Fine Chemicals, Schnelldorf, Germany). The determination of nitric oxide (NO) was performed with a total NO assay kit from R & D Systems (Minneapolis, MN, USA), which measures the more stable NO metabolites: NO_2_^−^ and NO_3_^−^ [23,24].

### 2.7. Western Blot Analysis

Protein expressions for NF-κB, p65 and cleaved caspase-3 were measured using Western blotting techniques. Lung tissues, harvested from the mice, were snap frozen immediately with liquid nitrogen and stored at −80 °C. Later, the tissues were weighed, rinsed with saline and homogenized with lysis buffer (pH 7.4), containing NaCl (140 mM), KCl (300 mM), trizma base (10 mM), EDTA (1 mM), Triton X-100 0.5% (*v*/*v*), sodium deoxycholate 0.5% (*w*/*v*), protease and phosphatase inhibitor. The homogenates were centrifuged for 20 min at 4 °C. The supernatants were collected and protein estimation was made using a Pierce bicinchoninic acid protein assay kit (Thermo Scientific, Waltham, MA, USA). A 35 µg sample of protein was electrophoretically separated by 10% sodium dodecyl sulfate polyacrylamide gel electrophoresis and then transferred onto polyvinylidene difluoride membranes. The immunoblots were then blocked with 5% non-fat milk and subsequently probed with either the rabbit monoclonal NF-κB p65 antibody (1:25,000 dilution, Abcam, Hong Kong, China) or rabbit monoclonal cleaved caspase-3 antibody (1:250 dilution, Cell Signalling Technology, Danvers, MA, USA), at 4 °C, overnight. The blots were then incubated with goat anti-rabbit IgG horseradish peroxidase conjugated secondary antibody (1:5000 dilution, Abcam), for 2 h, at room temperature, and developed using Pierce enhanced chemiluminescent plus Western blotting substrate Kit (Thermo Scientific). The densitometric analysis of the protein bands was performed for NF-κB p65 and caspase-3 with Typhoon FLA 9500 (GE Healthcare Bio-Sciences AB, Uppsala, Sweden). Blots were then re-probed with either mouse monoclonal GAPDH antibody (1:5000 dilution, Abcam) or mouse monoclonal β actin antibody (1:1000 dilution, Abcam) and used as a control.

### 2.8. DNA Damage Assessment by COMET Assay

Promptly after sacrifice, the lungs from control and DEP-exposed mice, with or without nootkatone pretreatment (*n* = 5 in each group), were removed. The COMET assay was performed, as described before [25,26], and the assessment of length of the DNA migration (i.e., diameter of the nucleus plus migrated DNA) was measured using image analysis Axiovision 3.1 software (Carl Zeiss, Toronto, ON, Canada) [27,28,29].

### 2.9. Statistics

All statistical analyses were performed with GraphPad Prism Software version 5. Comparisons between groups were performed by one way analysis of variance (ANOVA), followed by Newman–Keuls multiple range tests. All the data in figures were reported as mean ± SEM. *p*-Values < 0.05 are considered significant.

## 3. Results

### 3.1. Airway Hyperreactivity to Methacholine

**Figure 1:**
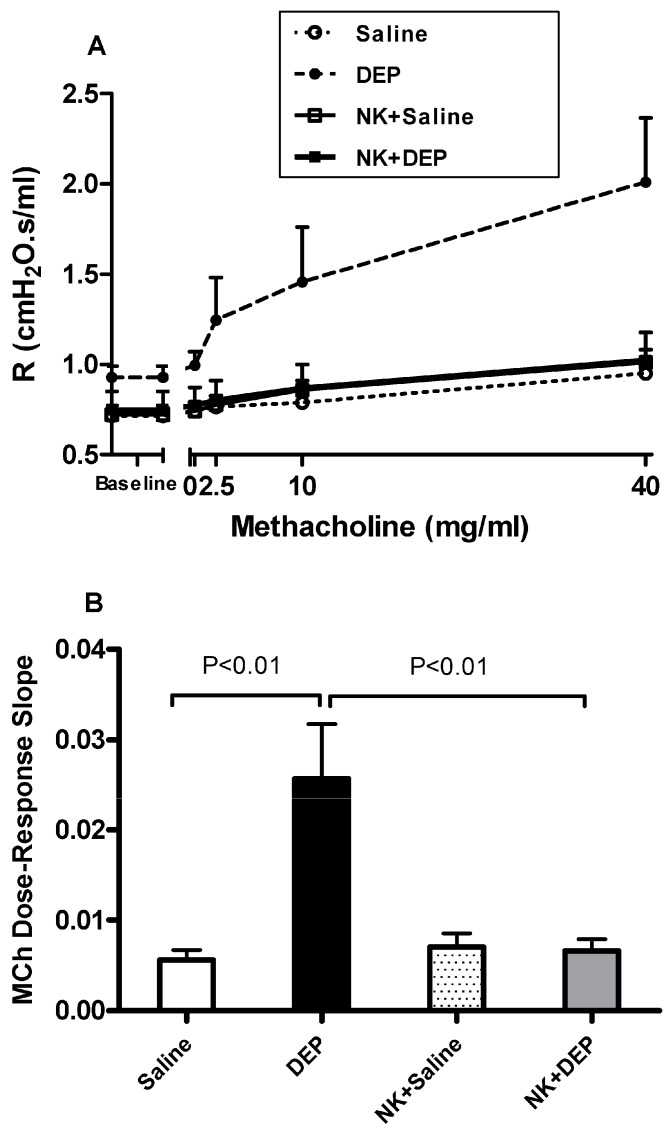
Airway hyper-responsiveness. The airway resistance (R), after increasing concentrations of methacholine (0–40 mg/mL), was measured via the forced oscillation technique (FlexiVent), 24 h after intratracheal instillation of either saline or diesel exhaust particles (DEP, 30 µg/animal), with or without nootkatone (NK) pretreatment. There was a dose–response relationship of total respiratory system resistance to increasing doses of MCh (**A**). From the resistance MCh dose–response curve in (**A**), an index of airway responsiveness was calculated as the slope of the linear regression, using 0–40 mg/mL concentrations (**B**). Data are mean ± SEM (*n* = 6–8).

The airway hyperreactivity to methacholine (0–40 mg/mL), measured by the forced oscillations technique, after i.t. instillation of either saline or DEP, with or without nootkatone pretreatment, is shown in Figure 1. I.t. instillation of DEP induced a dose-dependent and significant increase in airway resistance, compared with saline-instilled mice. No differences were noticed between saline and nootkatone+saline groups. Remarkably, nootkatone pretreatment induced a significant prevention of DEP-induced augmentation of airway resistance after increasing concentrations of methacholine (Figure 1A). The airway resistance to the methacholine dose–response curve was used to calculate an index of airway reactivity as the slope of the linear regression, using 0–40 mg/mL concentrations (Figure 1B). A significant augmentation of airway hyperreactivity to methacholine in mice i.t. instilled with DEP, compared with those instilled with saline (*p* < 0.01), and a complete abrogation of this effect, were observed following nootkatone pretreatment (*p* < 0.01) (Figure 1B).

### 3.2. Lung Histology

**Figure 2:**
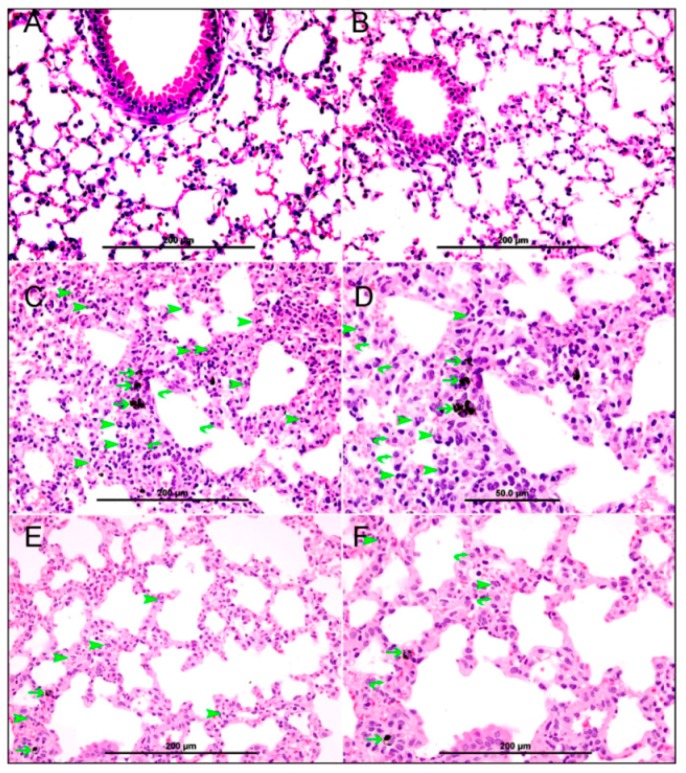
Representative light microscopy sections of lung tissues of mice, 24 h after administration of saline (**A**), nootkatone (NK)+saline (**B**), diesel exhaust particles (DEP; 30 µg/animal; **C**,**D**), and NK+DEP (**E**,**F**). (**A**,**B**) Both saline and NK+saline groups show normal lung tissue with unremarkable changes. (**C**,**D**) DEP-exposed lungs show particles within alveolar macrophages (thin arrows). There is severe expansion of the alveolar interstitial space with many neutrophil polymorphs (arrow head), and many macrophages (curved arrow). (**E**,**F**) The NK+DEP group shows DEP particles within alveolar macrophages (thin arrows). There is mild expansion of the alveolar interstitial space with a few neutrophil polymorphs (arrow head), and a few macrophages (curved arrow).

Light microscopy analysis of the lung sections obtained from saline-exposed mice displayed normal structures (Figure 2A). Likewise, lung sections from the nootkatone+saline group showed normal appearances and structures (Figure 2B). The lung sections of mice i.t. instilled with DEP showed particles inside alveolar macrophages which crossed to the alveolar interstitial space (Figure 2C,D). In this group, there was focal damage to the alveolar wall and severe expansion of the alveolar interstitial space, due to heavy neutrophil polymorphs and macrophage infiltration of the interstitium (Figure 2C,D). In the nootkatone+DEP group (Figure 2E,F), histological analysis of the lung showed the presence of DEP within the alveolar macrophages, which crossed to the alveolar interstitial space. There was a marked reduction in the inflammatory infiltrate (neutrophil polymorphs and macrophages) when compared with the DEP-exposed group. Moreover, the interstitial space was also reduced in size when compared with the DEP group.

### 3.3. Cell Composition and Number in BAL Fluid

**Figure 3:**
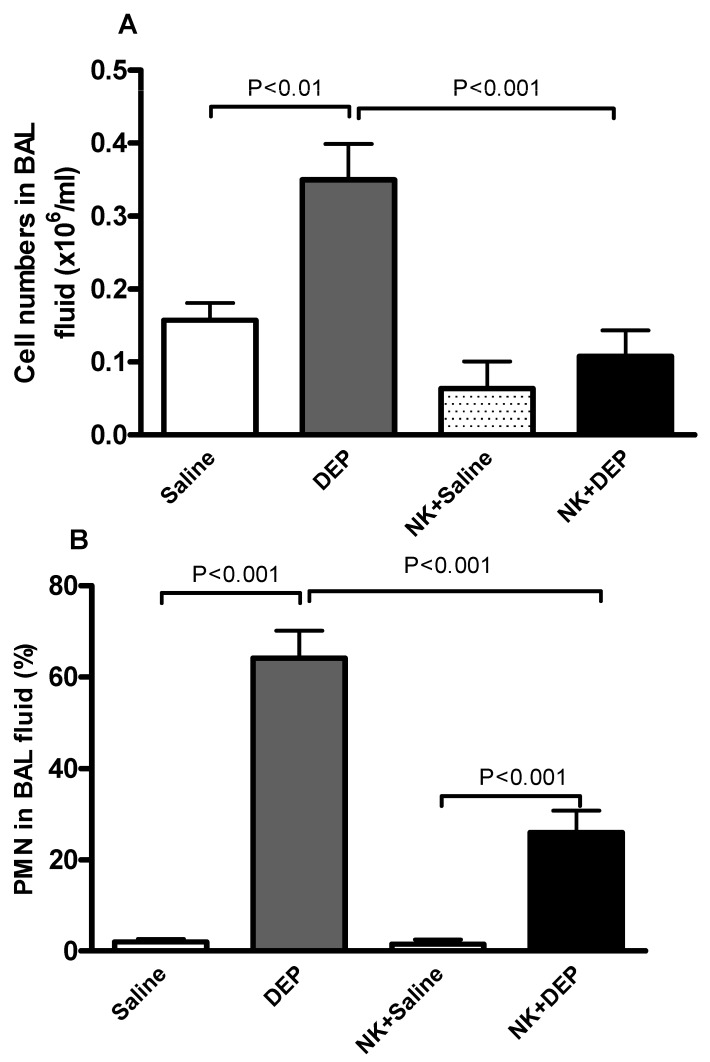
Number of cells (**A**) and polymorphonuclear neutrophils (PMN) (**B**) in bronchoalveolar lavage, 24 h after intratracheal instillation of either saline or diesel exhaust particles (DEP, 30 µg/animal), with or without nootkatone (NK) pretreatment. Data are mean ± SEM (*n* = 6–8 in each group).

The total number of cells and neutrophils in BAL fluid was significantly augmented by pulmonary exposure to DEP (Figure 3). The latter effects were significantly mitigated by nootkatone pretreatment (Figure 3).

### 3.4. 8-Isoprostane, GSH, Total NO and TNFα in Lung Homogenates

**Figure 4:**
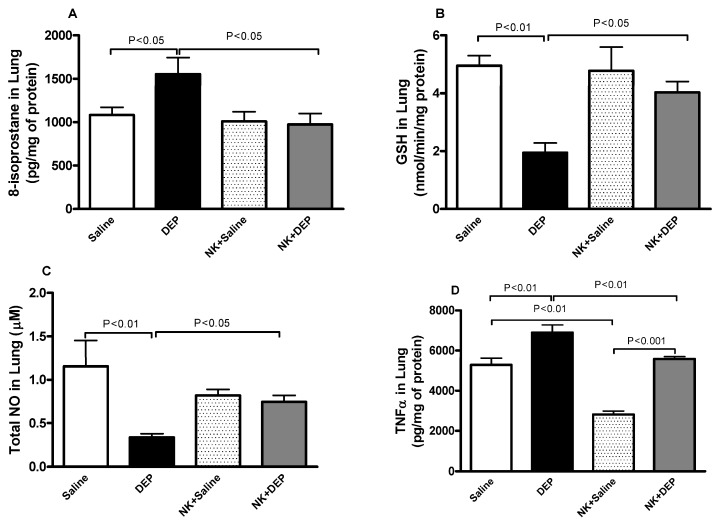
Lung homogenate levels of 8-isoprostane (**A**); reduced glutathione (GSH, **B**); total nitric oxide (NO, **C**) and tumor necrosis factor α (TNFα, **D**); 24 h after intratracheal instillation of either saline or diesel exhaust particles (DEP, 30 µg/animal) with or without nootkatone (NK) pretreatment. Data are mean ± SEM (*n* = 5–8 in each group).

As shown in Figure 4A–C, compared with the control group, 8-isoprostane, GSH and total NO levels in lung homogenates were significantly affected following DEP exposure.

DEP induced a significant increase of 8-isoprostane in the lung. Nootkatone pretreatment abrogated the effect of DEP and reduced the concentrations of 8-isoprostane to control values in the lung (Figure 4A).

DEP caused a significant decrease in the antioxidant, GSH, and this effect was significantly prevented by pretreatment with nootkatone (Figure 4B).

Likewise, total NO activity was decreased by pulmonary exposure to DEP. This effect was significantly mitigated by nootkatone pretreatment (Figure 4C).

TNFα concentration in lung homogenates was significantly augmented after DEP exposure compared with the control group (Figure 4D). TNFα concentration was significantly reduced in the nootkatone+DEP versus the DEP group (*p* < 0.01), and in the nootkatone+saline group versus the saline group (*p* < 0.001; Figure 4D).

### 3.5. Lung DNA Damage

**Figure 5:**
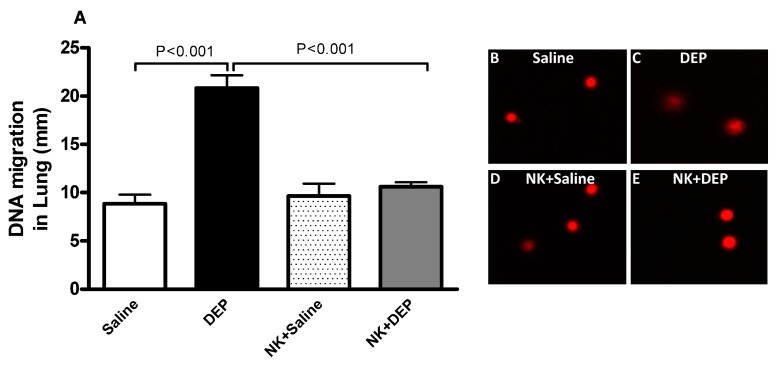
DNA migration (mm) in the lung tissues (**A**) evaluated by Comet assay, 24 h after intratracheal instillation of either saline or diesel exhaust particles (DEP, 30 µg/animal), with or without nootkatone (NK) pretreatment. Data are mean ± SEM (*n* = 5 in each group). Representative images, illustrating the quantification of the DNA migration by the Comet assay, under alkaline conditions, in control (**B**); DEP (**C**); NK+saline (**D**) and NK+DEP (**E**).

Figure 5 illustrates the impact of DEP exposure on lung DNA damage and the effect of nootkatone pretreatment therein. Compared with the control group, DEP exposure caused a significant increase in DNA migration (*p* < 0.001). Pretreatment with nootkatone significantly abated this effect (*p* < 0.001).

### 3.6. Western Blot Analysis for the Detection of Caspase-3 and NF-κB

**Figure 6:**
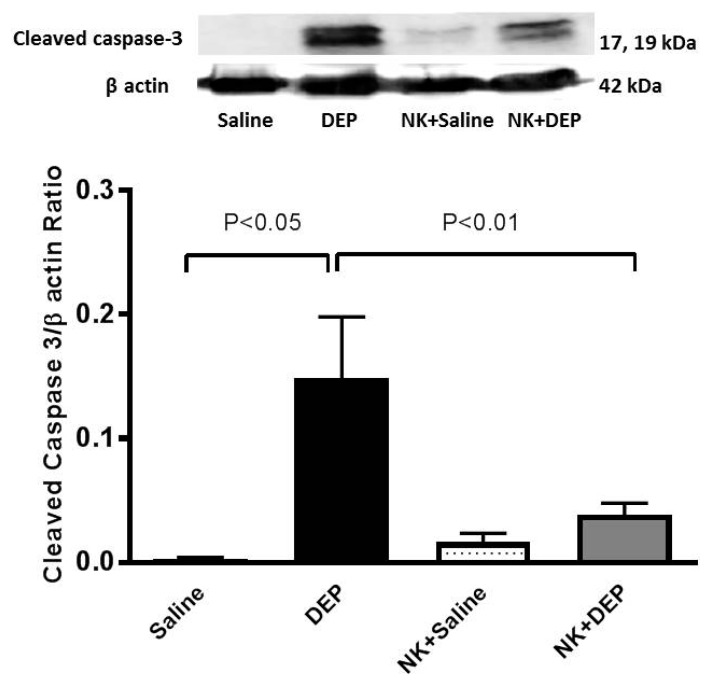
Cleaved caspase-3 levels in the lung tissues determined by Western blotting, 24 h after intratracheal instillation of either saline or diesel exhaust particles (DEP, 30 µg/animal), with or without nootkatone (NK) pretreatment. Data are mean ± SEM (*n* = 4 in each group).

The pulmonary exposure to DEP caused a significant increase in cleaved caspase-3 (*p* < 0.05. This effect was significantly prevented by nootkatone pretreatment (*p* < 0.01; Figure 6).

**Figure 7:**
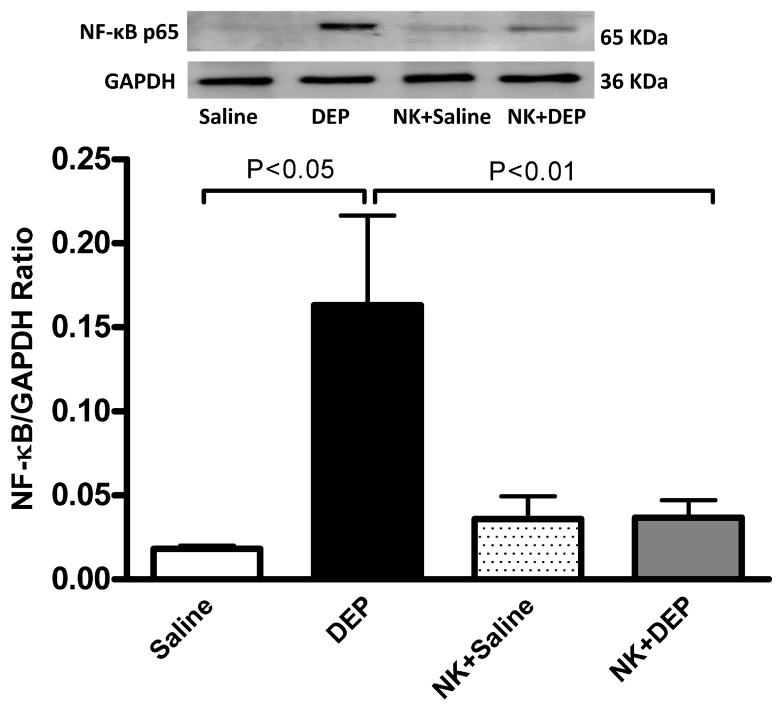
Nuclear factor-kappaB (NF-κB) levels in the lung tissues determined by Western blotting, 24 h after intratracheal instillation of either saline or diesel exhaust particles (DEP, 30 µg/animal), with or without nootkatone (NK) pretreatment. Data are mean ± SEM (*n* = 4 in each group).

Figure 7 shows that, compared with the control group, the i.t. instillation of DEP induced a significant increase in the expression of NF-κB (*p* < 0.05), and that the pretreatment with nootkatone significantly inhibited this effect (*p* < 0.01).

## 4. Discussion

In this work, nootkatone prevented a DEP-induced increase in airway resistance and pulmonary inflammation, oxidative stress, DNA damage and apoptosis. Moreover, nootkatone abrogated NF-κB activation in DEP-induced lung toxicity, suggesting that this compound exerts its protective effects by inhibiting the NF-κB activating pathway.

Human studies demonstrated a decrease in lung function, inflammation and oxidative stress, following short-term (hours to days) exposure to ambient particulate matter [30,31]. Thus, the current acute (24 h) study is relevant to human exposure scenarios. We studied the effect of 30 μg DEP/mouse, which is similar to doses studied previously in the context of ambient particulate matter exposure [32,33]. The United States Environmental Protection Agency stated a range of maximal city particulate matter concentrations, with aerodynamic diameter of less than 10 μm (PM_10_), as between 26 and 534 μg/m^3^ [34]. Numerous big cities in the world have much greater levels of PM_10_, with yearly averages of 200 to 600 μg/m^3^ and peak concentrations regularly surpassing 1000 μg/m^3^ [35]. Taking into consideration the highest value in the USA and supposing a minute ventilation of 6 l/min (~8.6 m^3^ over 24 h) for a healthy adult at rest, the total dose of PM inhaled over 24 h would be 4614 μg [32]. Human exposure to a daily dose of 4614 μg of PM would represent more than 35 μg of PM exposure for a mouse (25 g body weight), with a minute ventilation of 35–50 mL/min [32]. The dose we tested presently is similar to the comparative human dose of ±35 µg/mouse, reported by Mutlu et al. [32]. The pulmonary exposure to DEP was achieved by i.t. instillation, which represents a valid method of exposure because mice breathe through the nose, which filters the majority of inhaled particles [36,37].

We, and others, have previously reported that acute exposure to DEP induces lung inflammation in mice [8,15,38]. Likewise, human studies have shown the occurrence of lung inflammation and alteration of pulmonary function following acute exposure to DEP in healthy volunteers [5,7]. Nootkatone, naturally found in grapefruit oil, is currently used in flavor and fragrance applications [11]. Recent reports have suggested that nootkatone could be potentially used in insect control [11]. While the effects of nootkatone on allergy [39], obesity [12], platelet aggregation [40] and ischemia reperfusion [41] have been reported, its effects on lung injury induced by DEP have not been reported before.

Our study shows that DEP exposure induced a significant increase in airway hyperreactivity to methacholine, and that pretreatment with nootkatone abrogated this effect. Along with the increase in airway resistance, we found that nootkatone significantly reduced the influx of neutrophils in BAL fluid and the alveolar interstitial infiltration of macrophages and neutrophils assessed by histopathology. This effect is novel and expands the list of beneficial effects of this natural product. It has been reported that omega-3 polyunsaturated fatty acids protect against fine particle induced lung inflammation [42]. It has been shown also that emodin, an anthraquinone derivative from the Chinese herb, *Radix et Rhizoma Rhei*, mitigates DEP-induced lung inflammation and decreases lung function [9].

In order to assess the mechanism by which nootkatone exerts its protective effects on lung inflammation and airway resistance, we measured markers of oxidative stress and inflammation in lung homogenate. Our data show that nootkatone significantly prevented the increase in 8-isoprostane, a stable marker of lipid peroxidation which is generated by the peroxidation of arachidonic acid, catalyzed by free radicals [43]. Moreover, nootkatone significantly prevented the decrease in the GSH and NO, suggesting their consumption in the course of the breakdown of free radicals. It is well established that NO possesses oxygen radical scavenging properties, and that low levels of NO• are required to suppress lipid peroxidation [44]. Moreover, it has been reported that oxidative stress leads to reduced bioactivity of NO [45]. In fact, a decrease in NO following pulmonary exposure to nanoparticles has been reported in rats and mice [28,46,47]. The latter effect was explained by a significant decrease in NO production which coincided with the reduction of NO synthase activity and the excessive generation of reactive oxygen species [28,46,47]. The present findings suggest that the DEP induced oxidative stress in the lung, evidenced by an increase in 8-isoprostane and a decrease of the antioxidant GSH and the inhibitor of the lipid peroxidation chain reaction NO, could be mitigated by nootkatone, which exerts a potent antioxidant effect, by abrogating the increase in 8-isoprostane and replenishing the levels of GSH and NO in the lungs. Likewise, the increase in the pro-inflammatory cytokine TNFα was significantly reduced by nootkatone, compared with the DEP group. The concentration of TNFα in the nootkatone+saline group was even lower than that observed in the saline group. These effects suggest that nootkatone possesses anti-inflammatory and antioxidant properties. Nootkatone has been reported to improve the survival rate of septic mice through the induction of heme oxygenase-1, which has important antioxidant and anti-inflammatory effects [13]. The same authors also showed that nootkatone prevents the expression of inducible nitric oxide synthase and NO generation in RAW264.7 cells, following stimulation with lipopolysaccharide [13]. More recently, it has been reported that nootkatone inhibits TNFα/interferon γ-induced production of chemokines in HaCaT cells [48].

It has been suggested that ambient particulate matter and nanoparticles induce DNA oxidation injury resulting from oxidative stress and inflammation [28,29,49]. Here, we show that DEP exposure caused DNA damage assessed by COMET assay, and that this effect has been significantly inhibited by nootkatone pretreatment. It is well established that DNA damage can induce apoptosis [50]. The latter is the process of programmed cell death, which happens within the cells as a result of their shrinking, chromatin condensation, blebbing, nuclear fragmentation and chromosomal DNA fragmentation [50]. Caspases are key mediators of apoptosis. Among them, caspase-3 is a frequently activated death protease, catalyzing the specific cleavage of many key cellular proteins [51]. It is well established that reactive oxygen species can produce/regulate apoptosis, typically through caspase-3 activation [51]. We found that DEP induced a significant increase in cleaved caspase-3. Interestingly, we showed that nootkatone pretreatment significantly prevented the DEP-induced increase in cleaved caspase-3. It has been reported that eugenol, a methoxyphenol component of clove oil, with in vitro and in vivo anti-inflammatory and antioxidant properties, attenuated caspase-3 activation induced by DEP and prevented changes in lung mechanics, pulmonary inflammation, and alveolar collapse [52]. An in vitro study reported that essential oil from grapefruit, namely the dichloromethane fraction containing aldehyde compounds and nootkatone, induced apoptosis in human leukemic HL-60 cells [53]. The finding of the latter study is in disagreement with ours. This discrepancy could be related to the fact that we used pure nootkatone versus the use of the dichloromethane fraction containing aldehyde compounds and nootkatone, and the fact that our study has been performed in vivo as compared with the use of HL-60 cells [53].

To gain more insight into the mechanisms underlying the protective effects of nootkatone, we quantified NF-κB by Western blot. The latter has been implicated in the pathogenesis of a number of inflammatory diseases, including chronic obstructive pulmonary disease and asthma, and is crucial for activating the transcription of proinflammatory cytokines that cause inflammatory events, as well as oxidative stress and immunity [54]. Moreover, the activation of NF-κB has been shown to be associated with the onset of pulmonary inflammation following exposure to various environmental pollutants, including gaseous and particulate air pollution, cigarette smoke and engineered nanoparticles [4,55]. Activation of the NF-κB signalling pathway has been identified in human lung biopsies exposed to diesel exhaust [56] and in in vitro cell models [57]. Our data show that i.t. instillation of DEP stimulates NF-κB in the lung and the pretreatment with nootkatone significantly inhibited NF-κB activation. Our in vivo study corroborates an in vitro study, which showed that nootkatone inhibits TNFα/interferon γ-induced production of chemokines in HaCaT cells by inhibiting PKCζ and p38 MAPK signaling pathways that lead to activation of NF-κB [48]. Moreover, it has been shown that eugenol efficiently reduced LPS-induced lung toxicity by modulating pulmonary inflammation and remodeling, via a mechanism involving the prevention of TNF-α release and NF-κB activation [58]. It has also been demonstrated that targeting NF-κB could be an efficient therapeutic strategy for the treatment of septic shock, since the inhibition of NF-κB activation selectively inhibited the augmentation in inducible nitric oxide synthase expression (iNOS) activity and iNOS-mediated NO release [59]. At the cellular level, exposure to nanoparticles has been shown to activate several signaling pathways (i.e., NF-κB, NADPH oxidase) to coordinate pathophysiological responses, characterized by the production of inflammatory mediators and reactive oxygen species [4]. This is followed by a cascade of events that leads to the death of cells by different mechanisms (apoptosis, necrosis, autophagy) [4]. Additional work is needed to clarify the downstream chain signaling events involved in DEP induced lung toxicity, and the impact of nootkatone therein.

In conclusion, taken together, our data show that nootkatone pretreatment prevented lung inflammation assessed by BAL fluid analysis and histopathology and inhibited the production of TNFα and oxidative stress, and the subsequent DNA damage and apoptosis through a mechanism involving inhibition of NF-κB activation. The present study shows that in vitro anti-inflammatory antioxidant effects of nootkatone reported by others before [48] translate into an in vivo model of lung inflammation and could lead to possible prevention of lung toxicity, induced by ambient air pollution, and to new therapies for lung inflammatory diseases, in general.

## Supplementary Materials

The following are available online at http://www.mdpi.com/2072-6643/10/3/263/s1. Table S1: Pilot experiments used for the selection of nootkatone (NK) dose which was based on the assessment of cell numbers in bronchoalveolar lavage (BAL) fluid, 24 h after intratracheal instillation of either saline or diesel exhaust particles (DEP, 30 μg/animal) with or without pretreatment with various doses of NK (10, 30 or 90 mg/kg).

## References

[1] Atkinson R.W., Kang S., Anderson H.R., Mills I.C., Walton H.A. (2014). Epidemiological time series studies of PM_2.5_ and daily mortality and hospital admissions: A systematic review and meta-analysis. Thorax.

[2] Cai Y., Zhang B., Ke W., Feng B., Lin H., Xiao J., Zeng W., Li X., Tao J., Yang Z. (2016). Associations of Short-Term and Long-Term Exposure to Ambient Air Pollutants with Hypertension: A Systematic Review and Meta-Analysis. Hypertension.

[3] Steiner S., Bisig C., Petri-Fink A., Rothen-Rutishauser B. (2016). Diesel exhaust: Current knowledge of adverse effects and underlying cellular mechanisms. Arch. Toxicol..

[4] Nemmar A., Holme J.A., Rosas I., Schwarze P.E., Alfaro-Moreno E. (2013). Recent advances in particulate matter and nanoparticle toxicology: A review of the in vivo and in vitro studies. Biomed. Res. Int..

[5] Salvi S., Blomberg A., Rudell B., Kelly F., Sandstrom T., Holgate S.T., Frew A. (1999). Acute inflammatory responses in the airways and peripheral blood after short-term exposure to diesel exhaust in healthy human volunteers. Am. J. Respir. Crit. Care Med..

[6] Laumbach R.J., Kipen H.M., Ko S., Kelly-McNeil K., Cepeda C., Pettit A., Ohman-Strickland P., Zhang L., Zhang J., Gong J. (2014). A controlled trial of acute effects of human exposure to traffic particles on pulmonary oxidative stress and heart rate variability. Part. Fibre Toxicol..

[7] Xu Y., Barregard L., Nielsen J., Gudmundsson A., Wierzbicka A., Axmon A., Jonsson B.A., Karedal M., Albin M. (2013). Effects of diesel exposure on lung function and inflammation biomarkers from airway and peripheral blood of healthy volunteers in a chamber study. Part. Fibre Toxicol..

[8] Nemmar A., Al-Salam S., Zia S., Marzouqi F., Al-Dhaheri A., Subramaniyan D., Dhanasekaran S., Yasin J., Ali B.H., Kazzam E.E. (2011). Contrasting actions of diesel exhaust particles on the pulmonary and cardiovascular systems and the effects of thymoquinone. Br. J. Pharmacol..

[9] Nemmar A., Al-Salam S., Yuvaraju P., Beegam S., Ali B.H. (2015). Emodin mitigates diesel exhaust particles-induced increase in airway resistance, inflammation and oxidative stress in mice. Respir. Physiol. Neurobiol..

[10] Moon K.Y., Park M.K., Leikauf G.D., Park C.S., Jang A.S. (2014). Diesel exhaust particle-induced airway responses are augmented in obese rats. Int. J. Toxicol..

[11] Leonhardt R.H., Berger R.G. (2015). Nootkatone. Adv. Biochem. Eng. Biotechnol..

[12] Murase T., Misawa K., Haramizu S., Minegishi Y., Hase T. (2010). Nootkatone, a characteristic constituent of grapefruit, stimulates energy metabolism and prevents diet-induced obesity by activating AMPK. Am. J. Physiol. Endocrinol. Metab..

[13] Tsoyi K., Jang H.J., Lee Y.S., Kim Y.M., Kim H.J., Seo H.G., Lee J.H., Kwak J.H., Lee D.U., Chang K.C. (2011). (+)-Nootkatone and (+)-valencene from rhizomes of Cyperus rotundus increase survival rates in septic mice due to heme oxygenase-1 induction. J. Ethnopharmacol..

[14] Nemmar A., Al-Salam S., Beegam S., Yuvaraju P., Ali B.H. (2018). Thrombosis, systemic and cardiac oxidative stress and DNA damage induced by pulmonary exposure to diesel exhaust particles, and the effect of nootkatone thereon. Am. J. Physiol. Heart Circ. Physiol..

[15] Nemmar A., Al Salam S., Dhanasekaran S., Sudhadevi M., Ali B.H. (2009). Pulmonary exposure to diesel exhaust particles promotes cerebral microvessel thrombosis: Protective effect of a cysteine prodrug l-2-oxothiazolidine-4-carboxylic acid. Toxicology.

[16] Nemmar A., Al Maskari S., Ali B.H., Al Amri I.S. (2007). Cardiovascular and lung inflammatory effects induced by systemically administered diesel exhaust particles in rats. Am. J. Physiol. Lung Cell. Mol. Physiol..

[17] Nemmar A., Zia S., Subramaniyan D., Fahim M.A., Ali B.H. (2011). Exacerbation of thrombotic events by diesel exhaust particle in mouse model of hypertension. Toxicology.

[18] Nemmar A., Subramaniyan D., Zia S., Yasin J., Ali B.H. (2012). Airway resistance, inflammation and oxidative stress following exposure to diesel exhaust particle in angiotensin II-induced hypertension in mice. Toxicology.

[19] Nemmar A., Al-Salam S., Subramaniyan D., Yasin J., Yuvaraju P., Beegam S., Ali B.H. (2013). Influence of experimental type 1 diabetes on the pulmonary effects of diesel exhaust particles in mice. Toxicol. Lett..

[20] Nemmar A., Dhanasekaran S., Yasin J., Ba-Omar H., Fahim M.A., Kazzam E.E., Ali B.H. (2009). Evaluation of the direct systemic and cardiopulmonary effects of diesel particles in spontaneously hypertensive rats. Toxicology.

[21] Hardy R.D., Coalson J.J., Peters J., Chaparro A., Techasaensiri C., Cantwell A.M., Kannan T.R., Baseman J.B., Dube P.H. (2009). Analysis of pulmonary inflammation and function in the mouse and baboon after exposure to Mycoplasma pneumoniae CARDS toxin. PLoS ONE.

[22] Nemmar A., Al-Salam S., Beegam S., Yuvaraju P., Yasin J., Ali B.H. (2014). Pancreatic effects of diesel exhaust particles in mice with type 1 diabetes mellitus. Cell. Physiol. Biochem..

[23] Tsikas D. (2005). Methods of quantitative analysis of the nitric oxide metabolites nitrite and nitrate in human biological fluids. Free Radic. Res..

[24] Wennmalm A., Benthin G., Edlund A., Jungersten L., Kieler-Jensen N., Lundin S., Westfelt U.N., Petersson A.S., Waagstein F. (1993). Metabolism and excretion of nitric oxide in humans. An experimental and clinical study. Circ. Res..

[25] De Souza M.F., Goncales T.A., Steinmetz A., Moura D.J., Saffi J., Gomez R., Barros H.M. (2014). Cocaine induces DNA damage in distinct brain areas of female rats under different hormonal conditions. Clin. Exp. Pharmacol. Physiol..

[26] Olive P.L., Banath J.P., Fjell C.D. (1994). DNA strand breakage and DNA structure influence staining with propidium iodide using the alkaline comet assay. Cytometry.

[27] Hartmann A., Speit G. (1997). The contribution of cytotoxicity to DNA-effects in the single cell gel test (comet assay). Toxicol. Lett..

[28] Nemmar A., Yuvaraju P., Beegam S., Fahim M.A., Ali B.H. (2017). Cerium Oxide Nanoparticles in Lung Acutely Induce Oxidative Stress, Inflammation, and DNA Damage in Various Organs of Mice. Oxid. Med. Cell. Longev..

[29] Nemmar A., Yuvaraju P., Beegam S., Yasin J., Kazzam E.E., Ali B.H. (2016). Oxidative stress, inflammation, and DNA damage in multiple organs of mice acutely exposed to amorphous silica nanoparticles. Int. J. Nanomed..

[30] Hazucha M.J., Bromberg P.A., Lay J.C., Bennett W., Zeman K., Alexis N.E., Kehrl H., Rappold A.G., Cascio W.E., Devlin R.B. (2013). Pulmonary responses in current smokers and ex-smokers following a two hour exposure at rest to clean air and fine ambient air particles. Part. Fibre Toxicol..

[31] Rice M.B., Ljungman P.L., Wilker E.H., Gold D.R., Schwartz J.D., Koutrakis P., Washko G.R., O’Connor G.T., Mittleman M.A. (2013). Short-term exposure to air pollution and lung function in the Framingham Heart Study. Am. J. Respir. Crit. Care Med..

[32] Mutlu G.M., Green D., Bellmeyer A., Baker C.M., Burgess Z., Rajamannan N., Christman J.W., Foiles N., Kamp D.W., Ghio A.J. (2007). Ambient particulate matter accelerates coagulation via an IL-6-dependent pathway. J. Clin. Investig..

[33] Nemmar A., Al D.R., Alamiri J., Al H.S., Al S.H., Beegam S., Yuvaraju P., Yasin J., Ali B.H. (2015). Diesel Exhaust Particles Induce Impairment of Vascular and Cardiac Homeostasis in Mice: Ameliorative Effect of Emodin. Cell. Physiol. Biochem..

[34] Brook R.D., Franklin B., Cascio W., Hong Y.L., Howard G., Lipsett M., Luepker R., Mittleman M., Samet J., Smith S.C. (2004). Air pollution and cardiovascular disease—A statement for healthcare professionals from the expert panel on population and prevention science of the American Heart Association. Circulation.

[35] United Nations Environment Program, World Health Organization (WHO) (1994). Air Pollution in the world’s megacities. A Report from the U.N. Environment Programme and WHO. Environment.

[36] Driscoll K.E., Costa D.L., Hatch G., Henderson R., Oberdorster G., Salem H., Schlesinger R.B. (2000). Intratracheal instillation as an exposure technique for the evaluation of respiratory tract toxicity: Uses and limitations. Toxicol. Sci..

[37] Tamagawa E., Bai N., Morimoto K., Gray C., Mui T., Yatera K., Zhang X., Xing L., Li Y., Laher I. (2008). Particulate matter exposure induces persistent lung inflammation and endothelial dysfunction. Am. J. Physiol. Lung Cell. Mol. Physiol..

[38] Laks D., de Oliveira R.C., de Andre P.A., Macchione M., Lemos M., Faffe D., Saldiva P.H., Zin W.A. (2008). Composition of diesel particles influences acute pulmonary toxicity: An experimental study in mice. Inhal. Toxicol..

[39] Jin J.H., Lee D.U., Kim Y.S., Kim H.P. (2011). Anti-allergic activity of sesquiterpenes from the rhizomes of Cyperus rotundus. Arch. Pharm. Res..

[40] Seo E.J., Lee D.U., Kwak J.H., Lee S.M., Kim Y.S., Jung Y.S. (2011). Antiplatelet effects of Cyperus rotundus and its component (+)-nootkatone. J. Ethnopharmacol..

[41] Chang K.C., Lee D.U. (2016). Nootkatone from the rhizomes of *Cyperus rotundus* protects against ischemia-reperfusion mediated acute myocardial injury in the rat. Int. J. Pharm..

[42] Li X.Y., Hao L., Liu Y.H., Chen C.Y., Pai V.J., Kang J.X. (2017). Protection against fine particle-induced pulmonary and systemic inflammation by omega-3 polyunsaturated fatty acids. Biochim. Biophys. Acta.

[43] Yao H., Rahman I. (2011). Current concepts on oxidative/carbonyl stress, inflammation and epigenetics in pathogenesis of chronic obstructive pulmonary disease. Toxicol. Appl. Pharmacol..

[44] Hummel S.G., Fischer A.J., Martin S.M., Schafer F.Q., Buettner G.R. (2006). Nitric oxide as a cellular antioxidant: A little goes a long way. Free Radic. Biol. Med..

[45] Forstermann U. (2010). Nitric oxide and oxidative stress in vascular disease. Pflug. Arch..

[46] Nurkiewicz T.R., Porter D.W., Hubbs A.F., Stone S., Chen B.T., Frazer D.G., Boegehold M.A., Castranova V. (2009). Pulmonary nanoparticle exposure disrupts systemic microvascular nitric oxide signaling. Toxicol. Sci..

[47] Du Z., Zhao D., Jing L., Cui G., Jin M., Li Y., Liu X., Liu Y., Du H., Guo C. (2013). Cardiovascular Toxicity of Different Sizes Amorphous Silica Nanoparticles in Rats after Intratracheal Instillation. Cardiovasc. Toxicol..

[48] Choi H.J., Lee J.H., Jung Y.S. (2014). (+)-Nootkatone inhibits tumor necrosis factor alpha/interferon gamma-induced production of chemokines in HaCaT cells. Biochem. Biophys. Res. Commun..

[49] Moller P., Danielsen P.H., Karottki D.G., Jantzen K., Roursgaard M., Klingberg H., Jensen D.M., Christophersen D.V., Hemmingsen J.G., Cao Y. (2014). Oxidative stress and inflammation generated DNA damage by exposure to air pollution particles. Mutat. Res. Rev. Mutat. Res..

[50] Nowsheen S., Yang E.S. (2012). The intersection between DNA damage response and cell death pathways. Exp. Oncol..

[51] Savitskaya M.A., Onishchenko G.E. (2015). Mechanisms of Apoptosis. Biochemistry.

[52] Zin W.A., Silva A.G., Magalhaes C.B., Carvalho G.M., Riva D.R., Lima C.C., Leal-Cardoso J.H., Takiya C.M., Valenca S.S., Saldiva P.H. (2012). Eugenol attenuates pulmonary damage induced by diesel exhaust particles. J. Appl. Physiol..

[53] Hata T., Sakaguchi I., Mori M., Ikeda N., Kato Y., Minamino M., Watabe K. (2003). Induction of apoptosis by Citrus paradisi essential oil in human leukemic (HL-60) cells. In Vivo.

[54] Schuliga M. (2015). NF-kappaB Signaling in Chronic Inflammatory Airway Disease. Biomolecules.

[55] Traboulsi H., Guerrina N., Iu M., Maysinger D., Ariya P., Baglole C.J. (2017). Inhaled Pollutants: The Molecular Scene behind Respiratory and Systemic Diseases Associated with Ultrafine Particulate Matter. Int. J. Mol. Sci..

[56] Pourazar J., Mudway I.S., Samet J.M., Helleday R., Blomberg A., Wilson S.J., Frew A.J., Kelly F.J., Sandstrom T. (2005). Diesel exhaust activates redox-sensitive transcription factors and kinases in human airways. Am. J. Physiol. Lung Cell. Mol. Physiol..

[57] Totlandsdal A.I., Cassee F.R., Schwarze P., Refsnes M., Lag M. (2010). Diesel exhaust particles induce CYP1A1 and pro-inflammatory responses via differential pathways in human bronchial epithelial cells. Part. Fibre Toxicol..

[58] Magalhaes C.B., Riva D.R., DePaula L.J., Brando-Lima A., Koatz V.L., Leal-Cardoso J.H., Zin W.A., Faffe D.S. (2010). In vivo anti-inflammatory action of eugenol on lipopolysaccharide-induced lung injury. J. Appl. Physiol..

[59] Liu S.F., Ye X., Malik A.B. (1997). In vivo inhibition of nuclear factor-kappa B activation prevents inducible nitric oxide synthase expression and systemic hypotension in a rat model of septic shock. J. Immunol..

