# Supplementary material for: In Vivo Protective Effects of Nootkatone against Particles-Induced Lung Injury Caused by Diesel Exhaust Is Mediated via the NF-κB Pathway
Source: Nutrients. 2018 Feb 26;10(3):263. doi: 10.3390/nu10030263 (PMC5872681; doi:10.3390/nu10030263)
Supplement: Supplementary file 1 [file nutrients-10-00263-s001.docx]

**Supplementary Table S1.** Pilot experiments used for the selection of nootkatone (NK) dose which was based on the assessment of cell numbers in bronchoalveolar lavage (BAL) fluid, 24 h after intratracheal instillation of either saline or diesel exhaust particles (DEP, 30 µg/animal) with or without pretreatment with various doses of NK (10, 30 or 90 mg/kg).

| **Groups** | **Saline** | **DEP** | **NK10+Saline** | **NK10+DEP** | **NK30+Saline** | **NK30+DEP** | **NK90+Saline** | **NK90+DEP** |
| --- | --- | --- | --- | --- | --- | --- | --- | --- |
| Cell numbers in BAL fluid (x10^6^/ml) | 0.157±0.023 | 0.350±0.049**^*^** | 0.144±0.051 | 0.224±0.036 | 0.092±0.015 | 0.206±0.071 | 0.063±0.037 | 0.107±0.035**^**^** |

Data are mean ± SEM (n=5-8). *P<0.01 (Saline vs DEP) and **P<0.001 (DEP vs NK90+DEP).
